# Higher Tetanus Toxoid Immunity 2 Years After PsA-TT Introduction in Mali

**DOI:** 10.1093/cid/civ513

**Published:** 2015-11-09

**Authors:** Nicole E. Basta, Ray Borrow, Abdoulaye Berthe, Uma Onwuchekwa, Awa Traoré Eps Dembélé, Rachael Almond, Sarah Frankland, Sima Patel, Daniel Wood, Maria Nascimento, Olivier Manigart, Caroline L. Trotter, Brian Greenwood, Samba O. Sow

**Affiliations:** 1 Divisionof Epidemiology and Community Health, School of Public Health, University of Minnesota, Minneapolis; 2 Fogarty International Center, National Institutes of Health, Bethesda, Maryland; 3 Vaccine Evaluation Unit, Public Health England, Manchester Royal Infirmary, United Kingdom; 4 Centre pour le Développement des Vaccins, Ministère de la Santé, Bamako, Mali; 5 Faculty of Infectious and Tropical Diseases, London School of Hygiene and Tropical Medicine, United Kingdom; 6 Medical Research Council Unit, Fajara, The Gambia; 7 Disease Dynamics Unit, Department of Veterinary Medicine, University of Cambridge, United Kingdom

**Keywords:** meningococcal vaccines, tetanus, conjugate, seroprevalence, Africa

## Abstract

***Background.*** In 2010, mass vaccination with a then-new meningococcal A polysaccharide–tetanus toxoid protein conjugate vaccine (PsA-TT, or MenAfriVac) was undertaken in 1- to 29-year-olds in Bamako, Mali. Whether vaccination with PsA-TT effectively boosts tetanus immunity in a population with heterogeneous baseline tetanus immunity is not known. We assessed the impact of PsA-TT on tetanus toxoid (TT) immunity by quantifying age- and sex-specific immunity prior to and 2 years after introduction.

***Methods.*** Using a household-based, age-stratified design, we randomly selected participants for a prevaccination serological survey in 2010 and a postvaccination survey in 2012. TT immunoglobulin G (IgG) antibodies were quantified and geometric mean concentrations (GMCs) pre- and postvaccination among all age groups targeted for vaccination were compared. The probability of TT IgG levels ≥0.1 IU/mL (indicating short-term protection) and ≥1.0 IU/mL (indicating long-term protection) by age and sex was determined using logistic regression models.

***Results.*** Analysis of 793 prevaccination and 800 postvaccination sera indicated that while GMCs were low pre–PsA-TT, significantly higher GMCs in all age–sex strata were observed 2 years after PsA-TT introduction. The percentage with short-term immunity increased from 57.1% to 88.4% (31.3-point increase; 95% confidence interval [CI], 26.6–36.0;, *P* < .0001) and with long-term immunity increased from 20.0% to 58.5% (38.5-point increase; 95% CI, 33.7–43.3; *P* < .0001) pre- and postvaccination.

***Conclusions.*** Significantly higher TT immunity was observed among vaccine-targeted age groups up to 2 years after Mali's PsA-TT mass vaccination campaign. Our results, combined with evidence from clinical trials, strongly suggest that conjugate vaccines containing TT such as PsA-TT should be considered bivalent vaccines because of their ability to boost tetanus immunity.

In recent years, conjugate vaccines that link the polysaccharides on the outer surface of bacterial pathogens to protein carriers such as tetanus toxoid (TT) have been developed to protect against *Neisseria meningitidis*, *Haemophilus influenzae* type b (Hib), *Streptococcus pneumoniae*, and other pathogens [1]. Conjugate bacterial vaccines have markedly improved prevention and control efforts beyond results obtained with polysaccharide vaccines alone [2]. Conjugate vaccines have had significantly greater impact than polysaccharide vaccines because the presence of a carrier protein transforms the T-cell–independent immune response into a T-cell–dependent response, inducing immunologic memory and a strong immune response even among infants [3]. In addition, meningococcal, pneumococcal, and Hib conjugate vaccines have been shown to effectively prevent the acquisition of nasopharyngeal carriage of vaccine strains [2]. The 2010 introduction of an affordable group A meningococcal polysaccharide–TT protein conjugate vaccine, PsA-TT, or MenAfriVac, through mass vaccination campaigns targeting individuals aged 1–29 years in the African meningitis belt, has led to a dramatic decrease in group A meningococcal disease [4]. In addition to 10 µg of group A polysaccharide, PsA-TT contains 10–33 µg of TT [5], a level similar to other TT-containing vaccines. As a result, PsA-TT may boost immunity to tetanus in populations, adding benefit beyond prevention of meningococcal disease. Boosting tetanus immunity would be especially beneficial in resource-limited settings where tetanus incidence is highest and in areas where not all individuals have had prior exposure to sufficient doses of TT-containing vaccines to maintain immunity.

Currently, the World Health Organization (WHO) recommends that 3 doses of a TT-containing vaccine be administered in the first year of life as part of routine immunization programs with 2 booster doses of TT-containing vaccines given during childhood followed by vaccination of pregnant women to prevent neonatal tetanus [6]. Meeting the Global Vaccine Action Plan's (GVAP) target of 90% coverage nationally and >80% coverage with at least 3 doses of diphtheria-tetanus-pertussis vaccine in every district by 2015 remains a challenge for many low- and middle-income countries. By 2013, an estimated 66% of countries achieved the former target [7], and by 2012, 30% achieved the latter target worldwide [8]. Evidence suggests that 3 doses of TT-containing vaccine in the first year of life provides 3–5 years of protection, a booster dose between 4 and 7 years of age protects through adolescence, and another booster in adolescence protects through adulthood for 20–30 years [9]. However, vaccination schedules and vaccination coverage vary by country. In Mali, national immunization includes administration of pentavalent diphtheria, TT, pertussis, *H. influenzae*, and hepatitis B vaccine at 6, 10, and 14 weeks, with additional TT vaccination during pregnancy [10]. Mali does not administer adolescent TT boosters as part of its routine immunization program [10]. WHO-UNICEF estimates that in 2010, 82% of 12- to 23-month-olds in Mali received at least 1 dose of TT-containing vaccine, and 76% received all 3 recommended Expanded Programme on Immunization (EPI) doses [11].

Tetanus remains an ongoing challenge in some low- and middle-income countries [12], and an estimated 58 000 neonatal deaths due to tetanus occurred worldwide in 2010 [13]. Several countries located in the African meningitis belt, including Mali, Niger, Nigeria, and Chad had not eliminated maternal and neonatal tetanus as of mid-2014 [14]; only 11% of countries in the African region achieved both of the GVAP coverage goals by 2012 [15]. Enhanced protection against tetanus might be achieved as a result of vaccination with PsA-TT. To determine whether PsA-TT boosts tetanus immunity, we assessed the impact of the PsA-TT in Mali on population-level TT immunity by quantifying TT-specific IgG levels before and 2 years after the December 2010 PsA-TT mass vaccination campaign.

## METHODS

We conducted 2 cross-sectional serologic surveys in Mali's capital, Bamako, and villages nearby immediately prior to and 2 years after a PsA-TT mass vaccination campaign. The PsA-TT campaign was launched in December 2010 and targeted all individuals 1–29 years of age during a 4-week period in the south of the country, including the areas we surveyed. In January 2011, an evaluation of the PsA-TT campaign vaccination coverage level was undertaken in several districts of Bamako. Overall, 93% (95% confidence interval [CI], 92.6%–93.5%) of eligible participants were vaccinated with PsA-TT, with somewhat higher coverage both among children aged 1–4 years (97% of both males and females) and adolescents aged 5–14 years (98% of males and 96% of females) compared with adults aged 15–29 years (88% of males and 86% of females) [16].

### Prevaccination Serologic Survey

The prevaccination seroprevalence survey was conducted as part of the African Meningococcal Carriage Consortium (MenAfriCar) research program, which aimed to assess the impact of the 2010 introduction of PsA-TT on meningococcal carriage in Africa. MenAfriCar's methodologies have been detailed previously [17]. In brief, participants were selected randomly from the Djicoroni para district of Bamako and from Narena and Siby, neighboring villages to the southwest of Bamako, using a household-based, age-stratified sampling design, drawing from the approximately 78 000 residents in Djicoroni para identified through the Center for Vaccine Development (CVD)–Mali's demographic surveillance system and from the approximately 2000 residents identified through a census conducted in Narena and Siby for the purposes of this survey.

Participants were eligible if they were aged ≥6 months, had no acute or chronic illness, and if they or their parent or guardian provided consent. Participants responded to a questionnaire and provided up to 5 mL of blood.

### Postvaccination Serological Survey

The postvaccination seroprevalence survey was conducted as part of the US National Institutes of Health–funded PsA-TT (MenAfriVac) Antibody Persistence (MAP) study, which was designed to assess the impact of the 2010 introduction of PsA-TT on population-level immunity and the duration of protection against meningococcal disease provided by the vaccine [18]. Participants were randomly selected from the Banconi district of Bamako using a household-based, age-stratified sampling design drawing from the >130 000 residents identified through the CVD-Mali's demographic surveillance system. Participants were eligible if they were 1–29 years old during the 2010 PsA-TT campaign, were living in Banconi at the time of the campaign and at the time of the survey, had not previously been vaccinated with PsA-TT as a participant in a clinical trial, had no acute or chronic illness, and if they or their parent or guardian provided consent. Participants responded to a questionnaire and provided up to 8.5 mL of blood.

### Laboratory Methods

In both surveys, blood samples were collected and allowed to clot before being stored in a cool box (2°C–8°C). Samples were transported to the laboratory at CVD-Mali in Bamako within 4–6 hours where the serum was extracted, aliquoted, and stored at −20°C for the first survey and −80°C for the second survey prior to shipment on dry ice to the Vaccine Evaluation Unit, Public Health England (Manchester, United Kingdom) for TT IgG antibody level assessment.

Tetanus toxoid (National Institute for Biological Standards and Control [NIBSC], Hertfordshire, UK) was coupled to carboxylated microspheres (12.5 × 10^6^ microspheres/mL; Luminex, Texas) using a 2-step carbodiimide reaction [19, 20]. Carboxylated microspheres were activated in phosphate-buffered saline (PBS; pH 7.3) containing 5 mg/mL 1-ethyl-3(3-dimethylamino-propyl) carbodiimide-HCl and 5 mg/mL *N*-hydroxysulfosuccinimide (Sulpho-NHS; both Thermo Scientific, Cambridge, UK). Activated microspheres were then coupled to TT (1 mg/mL; NIBSC) and briefly vortex-mixed for 10 seconds, prior to incubation with shaking at 50 rpm at room temperature for 1.5 hours in the dark. Following this, the microspheres were washed 3 times with PBS (pH 7.3) by centrifugation (13 000 rpm for 2 minutes). On the final wash, microspheres were resuspended in 1 mL PBS and transferred to protein LoBind Eppendorf tubes (Eppendorf, Stevenage, UK) for storage in the dark at 4°C–8°C.

Tetanus toxoid–coupled beads were then used for the quantification of TT IgG antibodies using a fluorescent bead–based assay. A 10-point standard curve of 4-fold dilutions of human TE-3 standard reference serum (NIBSC; starting dilution 1/20) was prepared in serum diluent (1 × PBS containing 0.05% Tween 20 [Sigma-Aldrich, Dorset, UK] and 2% newborn bovine serum [Labtech International Ltd, East Sussex, UK]). Each dilution of standard, serum samples and quality control serum (25 µL of each) were diluted 1:100 in serum diluent and run in duplicate on the plate. A concentration of 5000 microspheres/region/well (25 µL) and test and control sera were incubated in a 96-well MV Multiscreen filter plate (Merck, Millipore, Massachusetts) for 20 minutes at RT with shaking at 500 rpm in the dark. Plates were washed twice by vacuum filtration and then 100 µL of a 1:200 dilution of R-phycoerythrin conjugated antihuman IgG (Stratech Scientific Ltd, Suffolk, UK) in PBS was added to each well and plates were incubated for 20 minutes with shaking (as previously, in the dark). Following another wash, the beads were resuspended in a final volume of 125 µL PBS-T and shaken for 5 minutes prior to reading on a Bio-Plex system (Bio-Rad, Hertfordshire, UK). Data were acquired using Bio-Plex Manager version 4 (Bio-Rad). Data for unknown test sera were generated from a 5-parameter logistic standard curve for the relevant reference serum and converted to international units (IU/mL).

### Statistical Analysis

In the prevaccination survey, field and laboratory data were recorded on specially designed paper forms and converted to an electronic format automatically using the TeleForm system (Teleform software version 10.4.1, HP Autonomy). In the postvaccination survey, field and laboratory data were entered directly into specially designed Microsoft Access (Microsoft Office) databases on small laptops. TT IgG data were reported in Microsoft Excel (Microsoft Office). All data were merged, managed, and analyzed using Stata SE 13.1 (StataCorp LP, College Station, Texas).

Age was defined as age at enrollment, which occurred between September and December 2010 for the prevaccination survey and age on 1 December 2010 for the postvaccination survey to assess cohort effects because we are interested in whether age-specific immunity, which would be expected to wane over time, was boosted by PsA-TT in cohorts of individuals targeted for PsA-TT vaccination. Age- and sex-specific pre- and postvaccination geometric mean concentrations (GMCs) and 95% CIs for TT IgG were calculated. To correct for the skewness of the distribution of TT IgG concentrations, the variable was log (base 10) transformed and differences were assessed using *t* tests on the log-transformed variable. Point estimates and CIs were back-transformed for presentation.

Data were analyzed to determine the proportion of participants before and after PsA-TT introduction with TT IgG response of ≥0.1 IU/mL and ≥1.0 IU/mL. The former is a marker of short-term immunity [9, 21], and individuals with concentrations above this threshold have evidence of protection against tetanus that is likely to wane within a year. However, cases have occurred in individuals with higher concentrations of antibody [21]. Therefore, TT IgG concentrations of ≥1.0 IU/mL are putative markers of long-term protection against tetanus that is likely to persist, and individuals with IgG levels in this range have evidence of recent boosting of tetanus immunity [21]. The 2-sample test of proportions was used to assess differences in the proportion protected within age–sex strata before and after vaccination. Multivariate logistic regression models were developed to investigate the association between age and sex with IgG levels at or above 0.1 IU/mL and 1.0 IU/mL, accounting for the clustered sampling design. The probability of short-term and long-term immunity by age and sex was predicted postestimation based on these models.

### Ethics Approval

The prevaccination study was approved by the ethics committees of the University of Bamako and the London School of Hygiene and Tropical Medicine. The postvaccination study was approved by the ethics committee of the University of Bamako and the institutional review boards at Princeton University and the University of Minnesota.

## RESULTS

In the prevaccination survey, 800 participants were enrolled between September and December 2010 prior to the mid-December 2010 PsA-TT mass vaccination campaign, which lasted approximately 4 weeks; sera from 793 of these participants was available for analysis. In the postvaccination survey, 800 participants were enrolled between December 2012 and January 2013, 2 years after the PsA-TT mass vaccination campaign, and sera from all participants were available for analysis. The proportion of females in the pre- and postvaccination surveys was similar (54% vs 57%), as was the mean household size (15 [range, 1–47] vs 16 [range, 2–50]), the distribution of the number of rooms per household (mean, 7.2 [range, 1–30] prevaccination vs mean, 6.8 [range, 1–30] postvaccination), and the average number of residents per room (2.5 vs 2.7) (Table 1). By design, half of the participants in the prevaccination survey lived in an urban area compared with all of the participants in the postvaccination survey (Table 1). There was no statistically significant difference between TT IgG GMCs (IU/mL) from urban and rural areas prevaccination (*P* = .30, 2 sample *t* test); these data are combined in the following analyses. All but 4 (0.5%) of the postvaccinated survey participants self-reported having received PsA-TT. Because we were interested in population-level changes among targeted age groups following the mass vaccination campaign, data from all participants, regardless of self-reported vaccination status, are included in the analyses.

**Table 1.** Characteristics of Participants in Each Survey

Characteristic	Prevaccination 2010		Postvaccination 2012	
	No.	%	No.	%
Age group^a,b^				
>1 y	31	4.0	…	…
1–2 y	59	7.6	50	6.3
3–5 y	115	14.8	151	18.9
6–10 y	107	13.8	200	25.0
11–17 y	123	15.9	200	25.0
18–29 y	141	18.2	199	24.9
>29 y	199	25.7	…	…
Sex^b^				
Females	416	54.1	457	57.1
Residence^c^				
Urban	394	49.7	800	100.0
Residents per household				
>15 people	311	40.6	335	41.9
Rooms per household				
>7	329	42.7	308	38.5

^a^ The age given is age at enrollment for 2010 survey and age as of 1 December 2010 for postvaccination survey.

^b^ Missing age and sex for 18 participants prevaccination.

^c^ Compared to living in a rural area.

### Changes in TT IgG Pre- and Postvaccination

**Figure 1.**
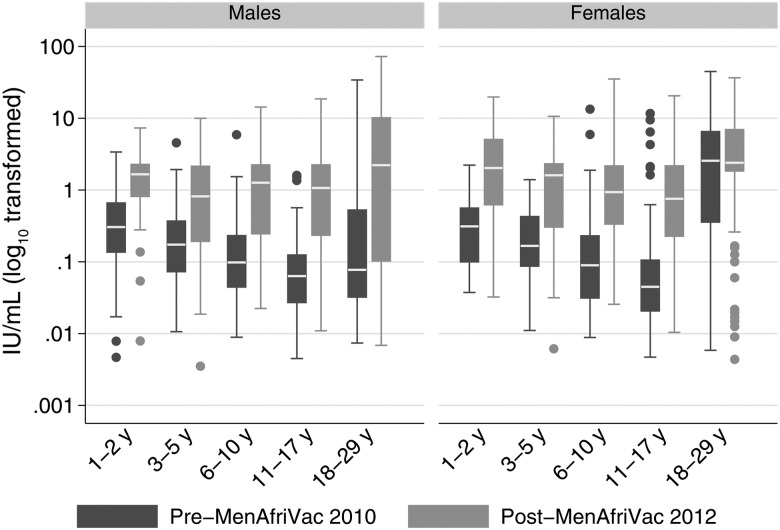
Box plot of tetanus toxoid immunoglobulin G (IU/mL) distribution before and after a meningococcal A polysaccharide–tetanus toxoid protein conjugate vaccine mass vaccination campaign. The age given is age at enrollment for 2010 survey and age as of 1 December 2010 for postvaccination survey.

The distribution of pre- and postvaccination GMCs by age and sex indicated that the minimum and maximum TT IgG concentrations observed were similar regardless of age, sex, or time of survey (Figure 1). Prevaccination, the GMC was significantly higher among females aged 1–29 years (0.26 [95% CI, .20–.33]) than males (0.13 [95% CI, .11–.16]) (*P* < .0001), with the highest prevaccination GMCs observed among 18- to 29-year-old women (Table 2). There were statistically significantly higher IgG GMCs in all age–sex strata 2 years postvaccination (Table 2). Postvaccination, the GMC point estimates among all females aged 1–29 years (1.13 [95% CI, .97–.1.31]) were higher than among males (0.82 ([95% CI, .68–1.00]), although the difference was not statistically significant (*P* = .1).

**Table 2.** Geometric Mean Concentrations of Tetanus Toxoid Immunoglobulin G (IU/mL) Before and After a Meningococcal A Polysaccharide–Tetanus Toxoid Protein Conjugate Vaccine Mass Vaccination Campaign by Sex and Age Group

Age Group	Males					Females				
	Prevaccination		Postvaccination			Prevaccination		Postvaccination		
	No.	GMC (95% CI)	No.	GMC (95% CI)	*P* Value^a^	No.	GMC (95% CI)	No.	GMC (95% CI)	*P* Value^a^
<1 y	16	.82 (.39–1.73)	…	…	…	15	.55 (.17–1.82)	…	…	…
1–2 y	35	.28 (.16–.47)	28	1.15 (.66–2.01)	.0004	24	.26 (.16–.41)	22	1.64 (.74–3.61)	.0001
3–5 y	63	.17 (.12–.23)	70	.68 (.46–.99)	<.0001	52	.17 (.12–.24)	81	.89 (.64–1.24)	<.0001
6–10 y	54	.12 (.08–.17)	106	.80 (.58–1.08)	<.0001	53	.09 (.06–.14)	94	.81 (.61–1.08)	<.0001
11–17 y	55	.07 (.05–.09)	84	.76 (.52–1.12)	<.0001	68	.06 (.04–.10)	116	.64 (.47–.86)	<.0001
18–29 y	42	.13 (.06–.26)	55	1.05 (.53–2.09)	.0001	99	1.46 (.97–2.22)	144	2.38 (1.83–3.11)	.04
>29 y	91	.06 (.04–.08)	…	…	…	108	.40 (.27–.61)	…	…	…

Data are shown as IU/mL.

Abbreviations: CI, confidence interval; GMC, geometric mean concentration.

^a^ *t* test.

### Percentage Putatively Protected Pre- and Postvaccination

**Figure 2.**
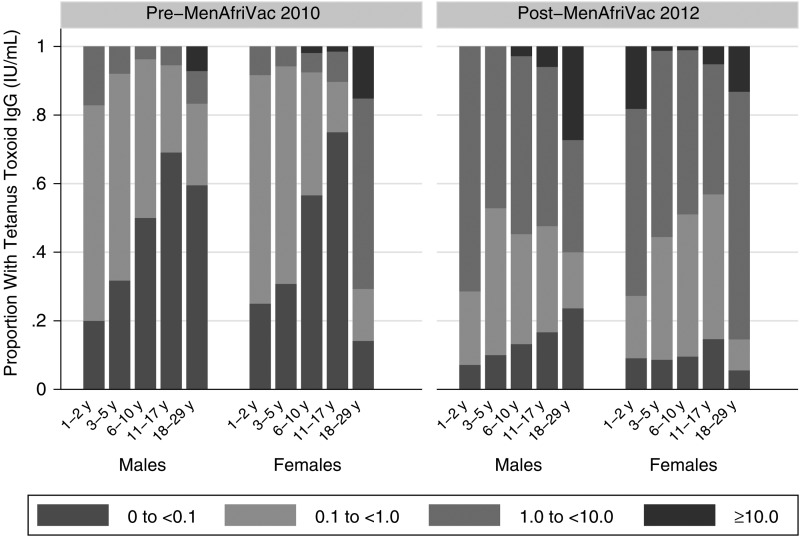
Tetanus toxoid immunoglobulin G (IgG) levels (IU/mL) by sex and age group before and after a Meningococcal A Polysaccharide–Tetanus Toxoid Protein Conjugate Vaccine mass vaccination campaign.

The percentage of participants with TT IgG levels indicative of short-term protection (≥0.1 IU/mL) increased from 57.1% prior to the vaccination campaign to 88.4% postvaccination, a 31.3-point increase (95% CI, 26.6–36.0; *P* < .0001; Table 3). The age- and sex-specific proportions of participants pre- and postvaccination with evidence of short-term and long-term TT immunity are shown in Figure 2. All age groups evidenced statistically significant changes in the percentage with TT IgG levels ­≥0.1 IU/mL 2 years after PsA-TT introduction (Table 3). Adolescents aged 11–17 years had the lowest proportion (27.6% [95% CI, 19.7%–35.5%]), with evidence of short-term protective levels of TT IgG pre–PsA-TT introduction and also the greatest percentage difference 2 years later (56.9-point increase [95% CI, 47.5–66.2]).

**Table 3.** Percentage Point Change in the Proportion of Individuals With Evidence of Short- and Long-term Anti-Tetanus Toxoid Immunity Before and After a Meningococcal A Polysaccharide–Tetanus Toxoid Protein Conjugate Vaccine Mass Vaccination Campaign

Age Groups	Percentage Point Change in Short-term (≥0.1 IU/mL) TT Immunity		Percentage Point Change in Long-term (≥1.0 IU/mL) TT Immunity	
	% (95% CI)	*P* Value	% (95% CI)	*P* Value
1–2 y	14.0 (1.1–27.0)	.0442	58.4 (43.2–73.6)	<.0001
3–5 y	22.0 (12.4–31.7)	<.0001	44.7 (35.5–53.9)	<.0001
6–10 y	41.8 (31.3–52.2)	<.0001	46.4 (38.2–54.8)	<.0001
11–17 y	56.9 (47.5–66.2)	<.0001	38.9 (30.4–47.3)	<.0001
18–29 y	17.1 (8.6–25.6)	<.0001	23.8 (13.8–33.8)	<.0001
Overall	31.3 (26.6–36.0)	<.0001	38.5 (33.7–43.3)	<.0001

Abbreviations: CI, confidence interval; TT, tetanus toxoid.

The proportion of participants with TT IgG levels indicative of long-term protection increased from 20.0% prior to the vaccination campaign to 58.5% postvaccination, a 38.5-point increase (95% CI, 33.7–43.3; *P* < .0001). All age groups experienced highly statistically significant changes in the percentage of participants who were putatively protected long-term 2 years after PsA-TT introduction (Table 3).

However, despite these increases, 2 years after PsA-TT introduction, 11.6% of participants still had TT IgG <0.1 IU/mL, indicating no evidence of tetanus protection, and 41.5% had TT IgG <1.0 IU/mL, indicating a lack of long-term protection.

### Multivariate Logistic Regression Analysis

The 1345 participants aged 1–29 years at the time of PsA-TT introduction were included in a multivariate logistic regression model. These participants were drawn from 625 households. The unadjusted odds of having a TT IgG level ≥0.1 IU/mL 2 years post–PsA-TT introduction vs prior was 5.7 (95% CI, 4.2–7.7; *P* < .0001), accounting for household clustering. After accounting for age group, sex, and their interaction (which was significant and thus retained in the model [Wald test *P* < .0001]), the adjusted odds of demonstrating short-term protection against TT 2 years after PsA-TT introduction vs prior was 7.3 (95% CI, 5.2–10.2; *P* < 0.001). The results for long-term protective concentrations ≥1.0 IU/mL were similar. The unadjusted odds of having a TT IgG level ≥1.0 IU/mL 2 years post–PsA-TT introduction vs prior was 5.6 (95% CI, 4.4–7.2, *P* < .0001), accounting for household clustering. After also accounting for age group, sex, and their interaction (which was again significant and retained in the model [Wald test *P* < .0001]), the odds of demonstrating long-term protection against tetanus 2 years after PsA-TT introduction vs prior was 9.0 (95% CI, 6.6–12.2; *P* < .0001).

**Figure 3.**
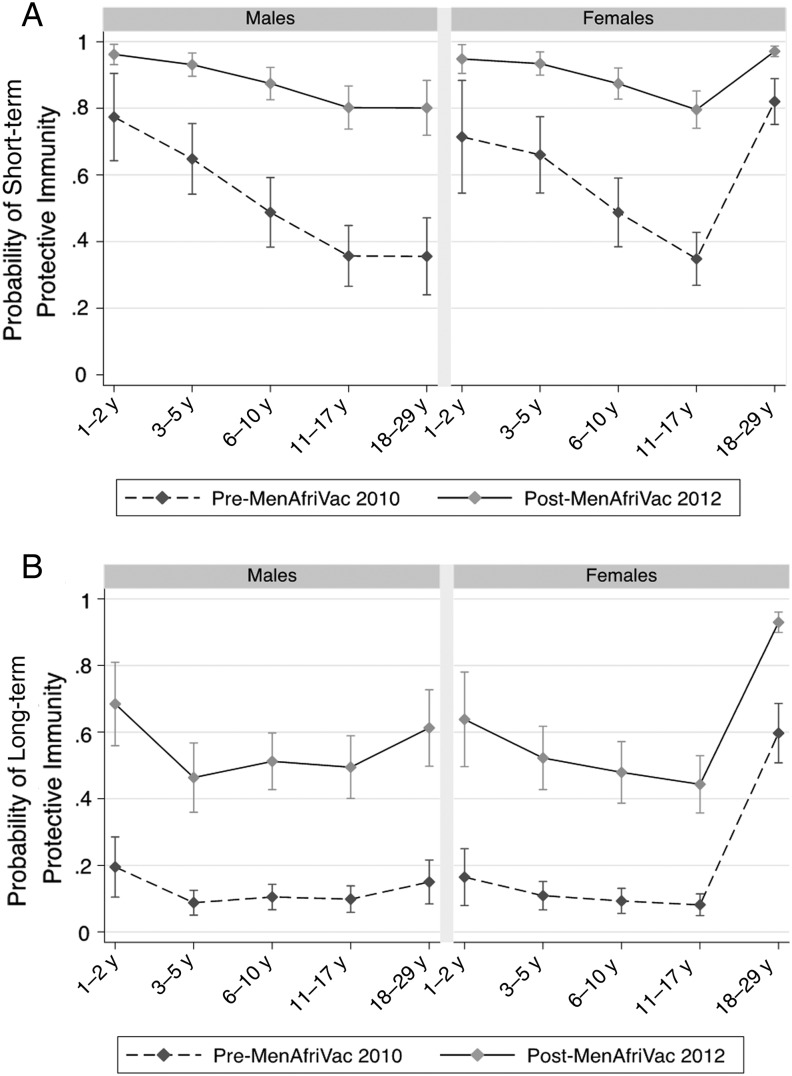
Predicted probability of short-term tetanus immunity (≥0.1 IU/mL) (*A*) and long-term tetanus immunity (≥1.0 IU/mL) (*B*) based on the adjusted logistic regression model by age group and sex before and after a Meningococcal A Polysaccharide–Tetanus Toxoid Protein Conjugate Vaccine mass vaccination campaign.

We also estimated the probability that an individual from each age–sex strata would have evidence of short-term and long-term immunity before and after PsA-TT introduction from the final models. In 2010, prior to PsA-TT introduction, both males and females <18 years of age had very similar age-specific probabilities of achieving short-term protection (Figure 3*A*) and long-term protection (Figure 3*B*). The probability of short-term protection decreased with increasing age across these groups prevaccination. The probability of both short-term and long-term protection was significantly higher among women compared with men aged 18–29 years. The probability of reaching both short-term and long-term protection was statistically significantly greater in all age–sex strata after the PsA-TT mass vaccination campaign (Figure 3).

## DISCUSSION

We found significantly higher TT immunity 2 years following the PsA-TT mass vaccination campaign in Mali across all age groups targeted for vaccination. Before PsA-TT introduction, TT immunity was low across all age groups, although higher in females than males and highest in women aged 18–29 years, who are likely to have received TT vaccination during pregnancy. Two years after PsA-TT introduction, TT GMCs and the proportion with both short-term and long-term protection against tetanus increased significantly across all groups targeted for PsA-TT vaccination. After accounting for age and sex, the odds of having at least short-term protection were 7.3 times higher and the odds of long-term protection were 9 times higher after PsA-TT introduction, indicating significant boosting of tetanus immunity in the population, which persisted 2 years after the campaign ended.

Our results are consistent with evidence from PsA-TT clinical trials that have demonstrated significant boosting of TT immunity among vaccine recipients 4 weeks after vaccination [22]. Criteria for inclusion in these clinical trials required participants to have completed all EPI vaccines; in contrast, in our study 42.8% (95% CI, 39.3%–46.3%) of participants demonstrated no evidence of tetanus immunity prior to PsA-TT introduction. Nevertheless, we found significant evidence of boosting of tetanus immunity over the 2-year study period.

A limitation of our study is that we were unable to obtain information about individual-level history of vaccination with TT-containing vaccines. Thus, we cannot be certain that the higher immunity observed after the vaccination campaign indicates an anamnestic response or whether it is indicative of a combination of anamnestic responses and prolonged response to primary immunization in some ages. A key question is whether the boosting of tetanus immunity observed is causally related to the introduction of PsA-TT or whether vaccination with other TT-containing vaccines could account for the increase in immunity observed. Examining our findings in light of routine immunization coverage data suggests that coverage with TT-containing vaccines remained relatively constant during this 2-year period and supports our claim that PsA-TT boosted tetanus immunity. WHO-UNICEF estimates that in 2010, 18% of children aged 12–23 months in Mali had received any doses of tetanus-containing vaccine and 24% had not received all 3 recommended EPI doses [11], which is consistent with our findings that 22% (95% CI, 12.3%–34.7%) of 1- to 2-year-olds sampled in the 2010 survey had TT IgG concentrations <0.1 IU/mL. In addition, estimated coverage in the first 2 years of life with 1 dose of TT-containing vaccine remained relatively constant, between 82% and 87% over the period 2002–2012 [11]. Some of the women in the reproductive age group who participated in this study would have received TT during pregnancy, so the increase in antibody concentrations seen in women of reproductive age at the population level following the vaccination campaign could be due primarily to an anamnestic response in subjects who had previously received at least 1 dose of TT. Therefore, it is possible that the significant increase in tetanus immunity among adult females is due to a combination of TT vaccination in pregnancy and PsA-TT. Individuals >1 year of age would not be eligible for TT-containing vaccines (unless targeted for vaccination during pregnancy) and, as there is no naturally acquired tetanus immunity, we can attribute the significant increases in tetanus immunity observed in young children and adolescents of both sexes and in adult males to the impact of PsA-TT. Overall, the proportion of the population with long-term immunity against tetanus remains low, which may be explained by limited exposure to the primary tetanus vaccination series historically in this population.

Our evidence suggests that polysaccharide-protein conjugate vaccines containing TT such as PsA-TT boost tetanus immunity and should be considered bivalent vaccines. Introduction of these vaccines in widespread population campaigns can help increase tetanus immunity across age groups not typically targeted for TT-containing vaccines or when coverage with TT vaccines is suboptimal. Recognition of this added benefit of PsA-TT could inform future vaccination strategies in resource-limited settings where maintaining high tetanus immunity, in addition to preventing meningococcal disease, is a priority.
